# Intention to receive vaccine against COVID-19 and associated factors among health professionals working at public hospitals in resource limited settings

**DOI:** 10.1371/journal.pone.0254391

**Published:** 2021-07-12

**Authors:** Mohammedjud Hassen Ahmed, Shuma Gosha Kanfe, Mohammedamin Hajure Jarso

**Affiliations:** 1 Department of Health Informatics, College of Health Sciences, Mettu University, Metu Zuria, Ethiopia; 2 Department of Psychiatry, College of Health Sciences, Mettu University, Metu Zuria, Ethiopia; ESIC Medical College & PGIMSR, INDIA

## Abstract

**Backgrounds:**

Health professionals are among the frontline of COVID-19 pandemic exposure and identified as a priority target group that need to receive COVID-19 vaccines. However, intention to receive vaccine is still matters the extent of COVID-19 vaccinations among health professionals. This study aimed to assess intention to receive COVID-19 vaccine and the factors that will determine their intention among health professionals working at public hospitals of Illu Aba Bora and Buno Bedelle zone hospitals.

**Methods:**

A cross-sectional study design was applied to assess the intention to receive COVID-19 vaccines among health professionals working in public health hospitals of Illu Aba Bora and Buno Bedelle zone hospitals. Self-administered questionnaire were used for assessing intention to receive COVID-19 Vaccine. Multiple linear regressions were performed to identify factors associated with intention to receive COVID-19 vaccine with p-value< 0.05 as cutoff point for statistical significance at 95% confidence interval (CI).

**Result:**

In this study, almost half of respondents 217(53.1% [95.0%: CI 49.3–58.9]) of study participants scored above the mean. Attitude (β = 0.54, 95% CI: [0.49, 0.63], p<0.01), knowledge (β = 0.27, 95% CI: [0.21, 0.35], p<0.01, perception (β = 0.43, 95% CI: [0.39, 0.56], p = 0.02 and age (β = 0.64, 95% CI: [0.51, 0.72], p<0.01 were variables associated with intention to receive vaccine against COVID-19.

**Conclusions:**

This study result indicated that the overall magnitude of intention to receive COVID-19 is low. increasing attitudes, knowledge and perception among health professionals related to COVID-19 vaccine will helps to increase the overall intention to receive vaccine against COVID-19.

## Background

The coronavirus disease 2019 (COVID-19) pandemic, being emerged in late December 2019 in Wuhan, China [1], infected millions of populations and causes several hundreds of thousands to be dead [2, 3]. As data reported from John Hopkins University Coronavirus Resource Center on August 13, 2020, The COVID-19 has affected more than seventeen million people across 215 countries and territories and caused deaths of more than 751.399 people [4]. The pandemic is still affecting the entire world though measurements to limit the spread of the disease by different stakeholders including governments [4, 5].

In the early stages of the outbreak, the COVID-19 has not gotten consideration even by being taken it as influenza (flu). Till declared as a pandemic, experts and politicians of over the world were not bothered of the pandemic (COVID-19) [6]. Efforts has been made to help to control the coronavirus disease (COVID-19) Pandemic targeting on development of vaccines against the COVID-19. As a result in Canada and European union, vaccines have been made and authorized in order to use it at the end of 2020 [7–9].

In Ethiopia, the Ministry of Health (MOH) and Ethiopian Public Health Institute (EPHI) in collaboration with partners have intensified response efforts to prevent the spread and severity of Corona Virus Disease 2019 (COVID-19) in Ethiopia. The national and the regional Public Health Emergency Operations Center (PHEOC) have been activated and laboratory diagnosis capacity has been expanded to other national institutions, subnational and private laboratories.

The national and regional PHEOC are playing a pivotal role in coordinating resources from different responding agencies and coordinating COVID-19 related information through regular EOC meetings and partners’ coordination forums. The MOH and EPHI are providing information to the public and stakeholders on a regular and uninterrupted manner using different means of communication modalities [10, 11]. According to data reported on February 23, 2021, total of 147,092 and 2,194 cases and death respectively were recorded [12]. With ongoing community transmission from asymptomatic individuals, disease burden is expected to rise. Healthcare workers (HCWs) are among the first group to receive vaccine. So, it is important to consider their intention about COVID-19 vaccination to better address barriers to widespread vaccination acceptance [13].

unintended towards COVID-19 vaccines and an uncertainty or unwillingness to receive vaccinations are major barriers to managing the COVID-19 pandemic in the long-term [14]. As a result, there will be an ongoing need for front-line health-care workers in patient-facing roles. Because of this work requires close personal exposure to patients with SARS-CoV-2, front-line health-care workers are at high risk of infection, contributing to further spread [15]. The pandemic COVID-19 has been affected the whole world and healthcare workers were accountable of the large number of infected groups. This is because health care workers can be exposed both as the victims of the disease and transmits it from infected to healthy person. Due to this reason, health care workers could be beneficial to themselves in addition to households and patients [16, 17].

Because of health care workers are among the first to receive the vaccines, their concerns about the safety of these vaccines must be addressed as early as possible [9, 17, 18]. Vaccination is central to controlling COVID-19. Its success relies on having safe and effective vaccines and also on high levels of uptake by the public over time. Addressing questions of population-level acceptability, stability of acceptance, and sub-population variation in acceptability are imperative [19]. This helps to experience the second wave of COVID-19 pandemic, high vaccination coverage by a safe and effective vaccine globally would be a great achievement.

Acceptance of vaccination among healthcare workers is mandatory to minimize and reduce the chain of transmission of COVID-19 [20]. The study conducted in USA stated that health care workers were planning to get vaccinated and the health organizations were delivering safe and effective vaccine [21]. The study [22] revealed that low intention to receive vaccines against COVID-19 was mostly driven by vaccine safety concerns. It has recommended that problems related to intention related to vaccine acceptances must be addressed before upcoming vaccination campaigns [16, 18, 22]. Uptake of any COVID-19 vaccine is an important challenge to be addressed. In a recent survey, more than one-third of lay respondents were unsure or did not intend to take the vaccine [13, 14, 19]. The study also identified that attitudes of health care workers could affect their intentions to receive vaccines against COVID-19 where 79.6% of health care workers has negative attitudes about vaccination [22]. As the study, general knowledge about vaccines, rejection of vaccines conspiracies, perceived severity of COVID-19 and risk factors for COVID-19, and politics were the significant predictors of intention to receive vaccines against COVD-19 [3]. The overall intentions to receive vaccines were very weak with 14.8% of respondents being unlikely to get vaccinated [3]. As the study shows, 60% of Americans would definitely or probably get a vaccine for the coronavirus [21]. About four-in-ten (39%) said that they are definitely or probably would *not* get a coronavirus vaccine. The study also shown as that 36% of respondents were willing to take the vaccine as soon as it became available but 56% of respondents were not sure or would wait to review more data [13].

However, the intention to receive COVID -19 in our setting remains as a gap and not assessed yet though the vaccine could be distributed as quickly as possible. Therefore, this study was conducted to assess the extent of intention to receive COVID-19 and its determinant factors among health care workers.

The case and death of corona virus is still increasing. To overcome this death, a lot of vaccines were being produced. The results of this study will help every stakeholders and health institutions including federal ministry of health to determine the amount and way of delivering vaccine services at the right time, with in the right people and at the right place. The study also helps to overcome shortcomings that could be occurred at the progress of this vaccination schedules.

## Materials and methods

### Study design, area, and period

A health-facility based cross-sectional study was conducted from January 2, to March 12, 2021 at Karl referral hospital, Darimu hospital, Dembi hospital, Chora hospital and Bedelle hospital, south west Ethiopia. The study was conducted in Ilu Aba Bore and Buno Bedelle zones. The two zones, Ilu Aba Bora and Buno Bedelle zones were together as Ilu Aba Bora zone till 2008 E.C when the later was established comprising the 10 eastern woredas of the late Ilu Aba Bora zone. Currently, Ilu Aba Bora (IAB) zone has 14 woredas and one administrative town. Ilu Aba Bora zone has forty-one (41) health centers, two (2) hospitals (one Referral hospital and one primary hospital). Buno Bedele zone has 10 woredas and one administrative town. The bedelle zone has three (3) hospitals called Buno Bedele general hospital, Dambi hospital and Chora hospitals. The health systems of both zones include hospitals, health centers and health posts. Mettu Karl Referral Hospital and Darimu hospitals provide primary and advanced health care service for IAB zones.

### Source and study populations

All health professionals working at all public hospitals of the two zones were considered as source and study population. All health professionals working at public health hospitals of the two zones and available during data collection time were included in the study. Health professionals who have less than 6 Months working experience from the two zones were excluded from the study due to new environment and temporary employee for the facility.

### Sample size determination and sampling procedure

A single population proportion formula, n = [(Zα/2)2P (1-P)]/d2 was used to calculate the sample size determination. Fifty(50) percent proportion was used to get the maximum sample size by taking into account 95% confidence interval (Zα/2 = 1.96), marginal of error (d) of 5% and 10% non-response. In line with the above consideration, the minimum final sample size was 423. As per our knowledge, there were no published data about intention to receive COVID-19 vaccine among health professionals. All five hospitals (Karl referral hospital, Darimu hospital, Dembi hospital, Chora hospital and Bedelle hospital) located within the selected zones were approached and used for this study. All health professionals in the selected hospitals were included in the study.

### Data collection

Quantitative data were collected using self-administered questionnaire. Questionnaire was prepared in English version. Eight data collectors who have good communication skill were recruited for data collection. Three health professionals who have experience on research work were supervised data collection process.

### Data processing and analysis

Data were entered in to Epi-info 7, exported to SPSS version 20 software. Descriptive statistics and univariate analysis was computed to describe socio demographic characteristics and magnitude of intention to receive COVID-19. A multiple linear regression analysis was computed to identify factors that independently influence the occurrence of the dependent variable. Variables which show significance at *P*-value of 0.2 during simple linear regression analysis were entered in to multiple linear regression analysis. The level of significance was determined at P-value of 0.05.

### Quality assurance

The questionnaire was pretested on 25 study participants who are working at Jimma referral hospital who were having similar demographic characteristics. Based on the result of the pretest, Necessary modifications of the questionnaire were done. The reliability of dependent and independent variable questions were checked, data collectors were trained and there was regular supervision.

### Ethics statement

The study protocol was reviewed and approved by ethical review board of Mettu University and Informed consent was obtained from each study participant. Permission letter also obtained from each Health facilities. Names of participants and other personal identifiers were not included in the data collection tool.

## Results

### Socio demographic characteristics

Out of the 423 questionnaires sent to the respondents, 409 participants were responded with a response rate of 96.7%. The mean age of the study subject was 33.4 with standard deviation of ±12. Nearly two-third 282(70.2%) of the participants were Male. Above half 221(54.0%) of the study participants were between the age of 30–39 and only 15 (3.7%) of them were greater than 59 years old. One hundred sixty three (39.9%) of the study participants were Nurses and 263(64.3%) of participants were degree. About 179(43.7%) of the study participants had between 5–10 years working experience, and only 18 (4.4%) had more than ten years working experience (Table 1).

**Table 1:** Socio-demographic characteristics of intention to receive vaccine against COVID-19 among health professionals at selected hospitals in south-west, Ethiopia, 2021.

Variables	Category	Frequency	Percent (%)
Sex	Male	287	70.2
	Female	122	29.8
Age	20–29	103	25.2
	30–39	221	54.0
	40–49	37	9.0
	50–59	33	8.1
	>59	15	3.7
Marital status	Unmarried	131	32.0
	Married	260	63.6
	Widowed	18	4.4
Profession	Nurse	163	39.9
	Psychiatry	7	1.7
	Optometry	6	1.5
	Midwifery	68	16.6
	Physician	105	25.7
	Health officer	6	1.5
	Anesthetics	7	1.7
	Laboratory	9	2.2
	Radiology	8	1.9
	Physiotherapy	7	1.7
	Pharmacist	17	4.1
	Other	6	1.5
Educational status	Diploma	102	24.9
	Degree	263	64.3
	MSc and above	44	10.8
Monthly Income	<5000	253	61.9
	5000–10000	129	31.5
	10000–15000	27	6.6
working experience	1–3	89	21.8
	3–5	123	30.1
	5–10	179	43.7
	>10	18	4.4
**Have you received all the necessary vaccines in your lifetime?**	Yes	276	67.5
	No	133	32.5

### Magnitude of intention to receive vaccine against COVID-19

In this study, almost half of respondents 217(53.1% [95.0%: CI 49.3–58.9]) of study participants scored above the mean. The mean score of intention to receive vaccine against COVID-19 was 11.3 with standard deviation of 2.7. The minimum and maximum score was 3 and 15 respectively.

### Frequency of intention to receive vaccine against COVID-19

One Hundred thirty six of respondents (33.2%) agreed that they were intended to receive vaccine against COVID-19. One hundred seven respondents (26.2%) were not predicted to receive COVID-19 vaccine in the near future as shown in Table 2.

**Table 2:** Intention to receive vaccine against COVID-19 among health professionals at selected hospitals in south-west, Ethiopia, 2021.

Items	Strongly disagree	Disagree	Neutral	Agree	Strongly agree
I intend to receive vaccine against COVID-19	101(24.7%)	70(17.1%)	23(5.6%)	136(33.2%)	79(19.3%)
I predict I will receive vaccine against COVID-19	107(26.2%)	68(16.6%)	28(6.8%)	129(31.5%)	77(18.8%)
I plan to receive vaccine against COVID-19	93(22.7%)	73(17.8%)	21(5.1%)	141(34.5%)	81(19.8%)

### Predictors associated intention to receive vaccine against COVID-19 among health professionals at selected hospitals in south-west, Ethiopia, 2021

Multiple linear regression analysis found that intention to receive vaccine against COVID-19 is predicted by attitude (β = 0.54, 95% CI: [0.49, 0.63], p<0.01), knowledge (β = 0.27, 95% CI: [0.21, 0.35], p<0.01, perception (β = 0.43, 95% CI: [0.39, 0.56], p = 0.02 and age (β = 0.64, 95% CI: [0.51, 0.72], p<0.01 as Table 3 shown below.

**Table 3:** Multiple linear regression result with estimates of intention to receive vaccine against COVID-19 among health professionals at selected hospitals in south-west, Ethiopia, 2021.

	Estimate	S.E.	C.R.	P	95% confidence interval	
					Lower	Upper
ATT → IU	0.54	0.01	10.30	***	0.49	0.63
KN → IU	0.27	0.05	8.32	***	0.21	0.35
PR → U	0.43	0.05	9.54	0.02	0.39	0.56
Age → IU	0.64	0.02	16.34	***	0.51	0.72
Income → IU	0.03	0.08	2.56	0.54	-0.91	0.21

The result of this study revealed that intention to receive vaccine against COVID-19 was influenced by attitude. One standard deviation additional change in attitude increases an intention to receive vaccine against COVID-19 by 0.54 standard deviations keeping other variables constant. Knowledge also influences intention to receive vaccine against COVID-19. One standard deviation additional change in knowledge increases an intention to receive vaccine by 0.27 standard deviations keeping other variables constant. Another predictor that influences intention to receive vaccine against COVID-19 was perception. With one standard deviation additional change in perception, an intention to receive vaccine against COVID-19 increases by 0.43 standard deviations keeping other variables constant. With one standard deviation additional change in age, an intention to receive vaccine against COVID-19 increases by 0.64 standard deviations keeping other variables constant.

Finally, attitude, knowledge, perception and age were positively associated with intention to receive vaccine against COVID-19 as shown below in the Table 3.

## Discussions

This study has attempted to discuss the intentions of health professionals to receive to COVID-19 vaccines. In order to support COVID-19 implementation, the study aimed at investigating an intention to receive the COVID-19 vaccines and those factors that determine their intentions to receive COVID-19 vaccine. Health professionals are among those frontline professionals those could be easily affected by COVID-19 pandemic. Throughout the world, health professionals has been serving as frontlines of pandemic response efforts, at high risk for occupational SARS-CoV-2 exposure and transmission, but they are almost universally identified as priority recipients of a forthcoming coronavirus vaccine [13, 21]. They also serve as trusted and influential stakeholders focusing on prevention and promotion of public health and have ultimately the role player of prescribing and administering of the approved coronavirus vaccines to their patients.

In this study, intention of health professionals to receive COVID-19 vaccines was 217(53.1% [95.0%: CI 49.3–58.9]). Almost more than half of study participants were intended to receive the COVID-19 vaccines as soon as the vaccines available. The result of this study was greater than the study conducted in USA which indicated that 36% of respondents were willing to take the vaccine as soon as it became available [13]. This variations might be due the fact that the period of data collections. This is because of information about COVID-19 has been quickly disseminated more than the time before. This study was also greater than the study conducted in China [23] where 40.0% of participants were intended to accept COVID-19 vaccination. Time variation and study design could cause these differences.

The study conducted in Hong Kong Chinese was also lower the result of this study where the prevalence of behavioral intentions of COVID-19 vaccination under the specific scenarios was very low and varied greatly (4.2% to 38.0%). The cause of the variation might be due to study populations and sample size [24]. However the result of this study was lower than the study conducted in French, 76.9% would accept a COVID-19 vaccine [25] and Scotland 77.6% [19] where 74% of study participants reported being willing to receive a COVID-19 vaccine in Italy [26]. The possible explanations for this variation could be the fact that the extent of COVID-19 prevalence. The prevalence of COVID-19 in France and Scotland was high when compared to that of in Ethiopia.

This study has also attempted to assess the factors that will determine an intention to receive COVID-19. Knowledge, age; attitudes and perception were significantly associated with intention to receive COVID-19. This study revealed that, an intention to receive COVID-19 vaccines could be determined by knowledge where, one standard deviation additional change in knowledge increases an intention to receive vaccine by 0.27 standard deviations keeping other variables constant with significant value of *p*<0.021.

The result of this study was supported by study conducted in USA and UK [3, 27, 28]. Another study also showed that low knowledge towards COVID-19 vaccine determines intention to receive COVID-19 vaccine which was similar with the result of this study [14]. This is because the targeted group will able to make their own decision about COVID-19 vaccine if they have gotten sufficient knowledge concerning the vaccine. Another study conducted stated that poor compliance with COVID-19 guidelines and low knowledge about COVID-19 predicted the extent of intention to receive vaccine that resulted in hesitancy and vaccine unwillingness [14].

According to this study, the extent of intention to receive COVID-19 was determined by attitudes of health professionals towards the vaccines. This is explained as; one standard deviation additional change in attitude increases an intention to receive vaccine by 0.54 standard deviations keeping other variables constant. This also to mean that negative attitude towards vaccine made intention to receive vaccine very low. Another study also revealed that negative attitudes towards vaccines were about 5 times higher relative risk of being unwilling to get a COVID-19 vaccine [14]. The study conducted in UK was also consistent with result of this study provided [27]. In addition to this, the result of this study was consistent with the study conducted in China [29], Italy [14] and UK [30].

This study also identified that an age is another determinant factors that will determine intention to receive COVID-19 vaccine. One standard deviation additional change in age, an intention to receive vaccine against COVID-19 increases by 0.64 standard deviations keeping other variables constant. This was consistent with the study conducted in French [16]. Age has been identified as the prominent factors that determines an intention to receive COVID-19 vaccine and it is consistent with study conducted in French [31], UK [27], Scotland [19] and USA [13].

The study revealed that perception was another predictor, with one standard deviation additional change in perception, an intention to receive vaccine against COVID-19 increases by 0.43 standard deviations keeping other variables constant. Similar studies including French [25] and China [24, 32]. These studies identified that, fear about COVID-19 and self-perceived risk of infection were associated with COVID-19 vaccine acceptance in health professionals.

## Conclusion

This study found that perception, knowledge, attitude and age all significantly impact health professional’s’ intentions to receive vaccine against COVID-19. This study has found significant factors that could determine intention to receive vaccine against COVID-19. So, to minimize the fear factor of COVID-19 vaccine, the ministry of health has to create awareness about the vaccine to increase intention to receive it.

The overall score of health professionals’ intention to receive vaccine against COVID-19 in our sample of south-west Ethiopia is moderate. So, the study has significant advantages for healthcare, policy makers and to the researchers in addition to federal ministry of health to enhance uptake of COVID-19 vaccine.

## Supporting information

S1 File (SAV)

S1 Questionnaire (DOCX)

## Abbreviations

ATT: Attitude
CI: Confidence Interval
EPHI: Ethiopian Public Health Institute
HCWs: HealthCare worker
IAB: ilu Aba Bora
KN: Knowledge
MoH: Ministry of Health
PHEOC: Public Health Emergency Operations Center
PR: Perception
WHO: World Health Organization
