# Supplementary material for: Intention to receive vaccine against COVID-19 and associated factors among health professionals working at public hospitals in resource limited settings
Source: PLoS One. 2021 Jul 12;16(7):e0254391. doi: 10.1371/journal.pone.0254391 (PMC8274862; doi:10.1371/journal.pone.0254391)
Supplement: S1 Questionnaire — (DOCX) [file pone.0254391.s002.docx]

For Data collectors use only:

Institution: _______________________ Questionnaire ID: ___________

**Questionnaire**

**Part 1:- Socio-demographic related Questions (encircle your responses)**

| **S.No** | **Questions** | **Responses** |  |
| --- | --- | --- | --- |
| 101 | Age in year | …………….. |  |
| 102 | Sex | 1. Male 2. Female |  |
| 103 | Marital status | 1. Unmarried status 2. Married |  |
| 104 | Educational status | 1. Certificate 2. Diploma 3. Degree 4. MSc and above |  |
| 105 | Monthly Income | ______________ |  |
| 106 | Religion | 1. Orthodox 2. Muslim 3. Protestant 4. Catholic 5. Other |  |
| 107 | Residence | 1. Rural 2. Urban |  |
| 108 | Your profession | 1. Health Officer 2. Medicine 3. Midwifery 4. Psychiatry 5. Physiotherapy 6. Optometry 7. Pharmacy 8. Laboratory 9. Nurse 10. Anesthesia 11. Radiology 12. Other please specify……….. |  |
| 109 | working experience in years | …………... |  |

**Have you received all the necessary vaccines in your lifetime?**

1. Yes
2. No

**Part 2:- Knowledge and attitude related Questions about intention to receive COVID 19 (encircle your responses)**

| **Part 3: Knowledge related questions on intention to receive Covid-19 vaccine** | | | | | | |
| --- | --- | --- | --- | --- | --- | --- |
| S. No | Please select the one to which your attitude is exactly matching, use the symbol in bracket to show your answer (X) | Strongly Disagree(**1**) | Disagree  (**2**) | Neutral  (**3**) | *Agree*  *(****4****)* | Strongly Agree  (**5**) |
| 301 | There is a presence of COVID-19 vaccine in Ethiopia | [1] | [2] | [3] | [4] | [5] |
| 302 | I came to know about COVID-19 vaccines first from social media | [1] | [2] | [3] | [4] | [5] |
| 303 | COVID-19 vaccine is very effective | [1] | [2] | [3] | [4] | [5] |
| 304 | **Overdose vaccine is very dangerous to use.** | [1] | [2] | [3] | [4] | [5] |
| 305 | Vaccination increase allergic reactions**.** | [1] | [2] | [3] | [4] | [5] |
|  | vaccination increase autoimmune diseases | [1] | [2] | [3] | [4] | [5] |

| **Part 3: Attitude related questions on intention to receive Covid-19 vaccine** | | | | | | |
| --- | --- | --- | --- | --- | --- | --- |
| S. No | Please select the one to which your attitude is exactly matching, use the symbol in bracket to show your answer (X) | Strongly Disagree(**1**) | Disagree  (**2**) | Neutral  (**3**) | *Agree*  *(****4****)* | Strongly Agree  (**5**) |
| 301 | **The newly discovered Covid-19 vaccines are safe.** | [1] | [2] | [3] | [4] | [5] |
| 302 | **I will take the COVID-19 vaccine without any hesitation, if it is available in** | [1] | [2] | [3] | [4] | [5] |
| 303 | **I will also encourage my family/friends/ relatives to get vaccinated**. | [1] | [2] | [3] | [4] | [5] |
| 304 | **It is not possible to reduce the incidence of COVID-19 without vaccination.** | [1] | [2] | [3] | [4] | [5] |
| 305 | **The COVID-19 vaccine should be distributed fairly to all of us.** | [1] | [2] | [3] | [4] | [5] |

| **Part 4: perception related Questions on intention to receive Covid 19** | | | | | | |
| --- | --- | --- | --- | --- | --- | --- |
| S. No | Please select the one to which your attitude is exactly matching, use the symbol in bracket to show your answer (X) | Strongly Disagree(**1**) | Disagree  (**2**) | Neutral  (**3**) | *Agree*  *(****4****)* | Strongly Agree  (**5**) |
| 301 | **I think that if everyone in the society maintains the preventive measures, the COVID-19 pandemic can be eradicated without Vaccination** | [1] | [2] | [3] | [4] | [5] |
| 302 | **I think the newly discovered Covid-19 vaccine may have side effects** | [1] | [2] | [3] | [4] | [5] |
| 303 | **I should have been vaccinated** | [1] | [2] | [3] | [4] | [5] |
| 304 | **I supposed health professionals to be vaccinated first.** | [1] | [2] | [3] | [4] | [5] |
| 305 | **I think the vaccine should be administered free of charge in Ethiopia.** | [1] | [2] | [3] | [4] | [5] |
|  | **I Would buy the vaccine at my own expense if it was not provided free by the government** | [1] | [2] | [3] | [4] | [5] |

| **Part 5: Intention related questions on Covid-19 vaccine** | | | | | | |
| --- | --- | --- | --- | --- | --- | --- |
| S. No | Please select the one to which your attitude is exactly matching, use the symbol in bracket to show your answer (X) | Strongly Disagree(**1**) | Disagree  (**2**) | Neutral  (**3**) | *Agree*  *(****4****)* | Strongly Agree  (**5**) |
| 501 | **I intend to receive Covid 19 vaccine** | [1] | [2] | [3] | [4] | [5] |
| 502 | **I predict I will receive Covid 19 vaccine** | [1] | [2] | [3] | [4] | [5] |
| 503 | **I plan to receive covid 19 vaccine** | [1] | [2] | [3] | [4] | [5] |
